# Delayed spontaneous remission of acquired factor V inhibitor refractory to immunosuppressive therapy with pregnancy-associated improvement

**DOI:** 10.3389/pore.2023.1611250

**Published:** 2023-06-02

**Authors:** Andrea Ceglédi, János Dolgos, Mónika Fekete, László Gopcsa, Andrea Várkonyi, Beáta Vilimi, Gábor Mikala, Imre Bodó

**Affiliations:** ^1^ Departments of Hematology and Stem Cell Transplantation, South Pest Central Hospital, National Institute of Hematology and Infectious Diseases, Saint Ladislaus Campus, Budapest, Hungary; ^2^ Department of Public Health, Semmelweis University, Budapest, Hungary; ^3^ Department of Internal Medicine and Hematology, Semmelweis University, Budapest, Hungary; ^4^ Department of Hematology and Medical Oncology, Emory University, Atlanta, GA, United States

**Keywords:** factor V inhibitor, coagulopathy, gestation, bleeding disorder, autoimmune

## Abstract

**Introduction:** Acquired factor V inhibitor (AFVI) is a rare autoimmune bleeding disorder. The treatment of AFVI is challenging, and patients often require both bleeding control and inhibitor eradication.

**Methods:** We conducted a retrospective analysis of the medical records of a 35-year-old Caucasian woman who presented with severe AFVI-induced bleeding and subsequent immunosuppressive therapy.

**Results:** To provide haemostasis, rFVIIa was given with good efficacy. The patient was treated with various combinations of immunosuppressive regimens over the course of 2.5 years, including plasmapheresis plus immunoglobulins, dexamethasone + rituximab, cyclophosphamide + dexamethasone + rituximab + cyclosporine, cyclosporin + sirolimus + cyclophosphamide + dexamethasone, bortezomib + sirolimus + methylprednisolone, and sirolimus + mycophenolate mofetil. Although these treatment modalities resulted in intermittent partial reversals of AFVI over 2.5 years, eventually the inhibitor became therapy-resistant. However, following the discontinuation of all immunosuppressive therapy, the patient experienced a partial spontaneous remission, which was followed by a pregnancy. During the pregnancy, the FV activity increased to 54% and the coagulation parameters returned to normal levels. The patient underwent Caesarean section without any bleeding complications and delivered a healthy child.

**Discussion:** The use of an activated bypassing agent for bleeding control is effective in patients with severe AFVI. The presented case is unique because the treatment regimens included multiple combinations of immunosuppressive agents. This demonstrates that AFVI patients may undergo spontaneous remission even after multiple courses of ineffective immunosuppressive protocols. Additionally, pregnancy-associated improvement of AFVI is an important finding that warrants further investigation.

## Introduction

Acquired factor V deficiency is a rare hemostatic disorder caused by antibodies against factor V, resulting in varying degrees of hemorrhagic manifestations. Approximately 200 cases of this rare acquired coagulopathy have been reported in the literature [1–32]. The incidence of acquired factor V deficiency is estimated to be between 0.06 and 0.09 per one million people per year [33], although the true incidence may be somewhat higher because of undiagnosed oligosymptomatic/asymptomatic cases. The antibodies neutralize and facilitate clearance of factor V. In routine clinical practice diagnosis is usually made based on the demonstration of a prolongation of both the prothrombin time (PT) and activated partial thromboplastin time (aPTT), normal thrombin time and fibrinogen level. In mixing studies, the addition of normal plasma does not fully correct the prolongations. The diagnosis requires the measurement of factor V activity and the factor V inhibitor (Bethesda titer) assay, which is usually performed using a prothrombin time-based system. The clinical manifestation of this coagulopathy ranges from asymptomatic laboratory findings to life-threatening bleeding. There is a range of diverse clinical conditions that have been shown to associate with the presence of acquired factor V inhibitors (AFVI), including exposure to topical bovine thrombin glue during surgical procedures [4, 8, 16, 24, 27], infections [1, 10, 29] including COVID-19 [6, 11] and HIV [7], use of antibiotics (including post-operative antibiotic usage) [15, 34], malignancies [10, 18, 35] (including multiple myeloma [3, 5]), treatment with anti-cancer drugs [13], other drug treatments [14], autoimmune diseases [20], and transfusion. 20% of the AFVI cases are idiopathic. Here, we analyse a case of severe AFVI-induced bleeding that proved refractory to multiple immunosuppressive regimens. However, spontaneous remission occurred several years after discontinuation of therapy, and the patient went on to have a successful pregnancy. This study highlights the complexity of managing AFVI and the possibility of spontaneous remission, even after multiple courses of ineffective immunosuppressive protocols.

## Methods

We conducted a retrospective analysis of the medical records of a 35-year-old Caucasian woman who presented with severe AFVI-induced bleeding and subsequent immunosuppressive therapy. The publication of this report was approved by the ethics committee of the South Pest Central Hospital/National Institute of Hematology and Infectious Diseases, and the patient provided written informed consent.

Data were collected from the patient’s medical records, including laboratory test results, imaging studies, surgical reports, and medication administration records. The patient’s clinical course was tracked from the initial admission to our department on 21st February 2014, to the present day. Descriptive statistics were used to analyze the patient’s clinical data, including laboratory test results and medication regimens. The time course of changes in coagulation parameters, including FV activity, was depicted graphically. The response to therapy was assessed by monitoring the patient’s bleeding symptoms, laboratory test results, and adverse events.

Limitations of this retrospective analysis include the potential for missing or incomplete data and the inability to establish causality between the patient’s clinical course and the treatments administered. Furthermore, this case report describes the experience of a single patient and may not be generalizable to other patients with similar conditions.

## Results

On 21st February 2014, a 35-year-old Caucasian woman was admitted to our department for extensive postoperative bleeding after laparascopic gynecological surgery for an ovarian cyst. The preoperative records did not include information about hemostasis. A 4 cm abdominal hematoma required reoperation and right adnexectomy. Histology showed a benign ovarian cyst. The patient had excessive intraoperative bleeding and her hematocrit fell to 0.14, hemoglobin to 44 g/L, despite receiving fresh frozen plasma (FFP) and red blood cell transfusion. Gastrointestinal bleeding (melaena) also developed. The patient was transferred for management of critical bleeding.

The patient had an unremarkable past medical history and had no prior bleeding history in spite of a Cesarean section 5 years earlier. She denied taking any medications or nutritional supplements. There was no family history of bleeding. She worked as an accountant. A physical examination on admission revealed an afebrile and hemodynamically stable patient.

**FIGURE 1:**
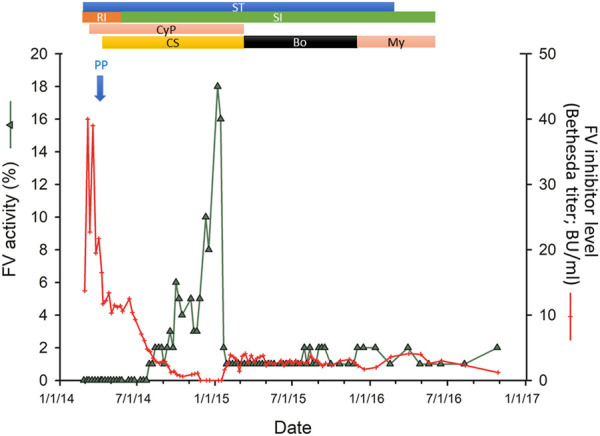
Time course of immunosuppressive therapies. Changes in the factor V (FV) inhibitor levels (Bethesda titer) in response to the immunosuppressive treatment regimens used (red) are shown as a function of time. Concomitant changes in FV activity are superimposed (green). Bars indicate the administration of combination immunosuppressive therapies. Note that immunosuppressive therapies were discontinued from June 2016. The subsequent spontaneous reversal of AFVI is shown in Figure 2. ST, corticosteroid treatment; RI, rituximab; SI, sirolimus; CyP, cyclophosphamide; CS, cyclosporin; Bo, bortezomib; My, mycophenolate mofetil; PP, Plasmapheresis.

**FIGURE 2:**
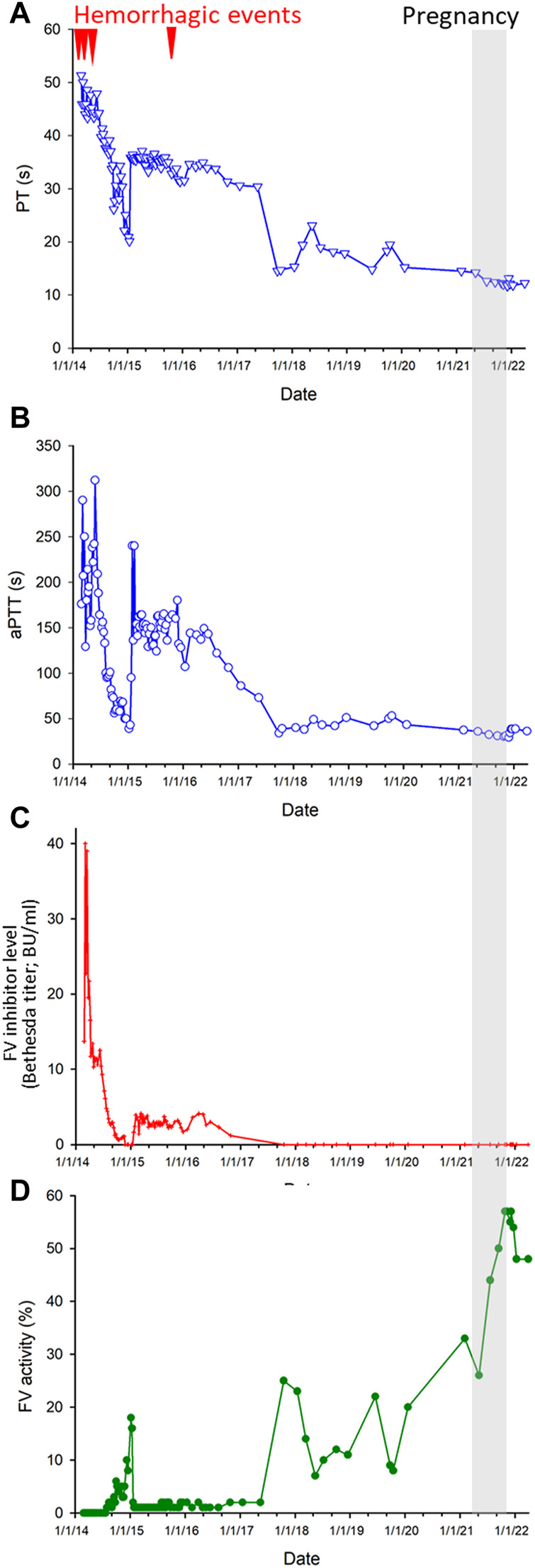
Time course of spontaneous reversal of AFVI. **(A)** Changes in prothrombin time (PT) as a function of time. **(B)** Changes of activated partial thromboplastin time (APTT) as a function of time. **(C)** Changes in the factor V inhibitor levels (Bethesda titer) as a function of time. **(D)** Changes of factor V activity as a function of time. Note that immunosuppressive therapies were discontinued from June 2016. Arrowheads: major hemorrhagic events.

Laboratory analysis showed severe anemia, hematocrit of 0.14, a moderate dilutional thrombocytopenia of 74 G/L and, and normal white blood cell count. Electrolyte and creatinine levels, liver function test results were normal. PT was: 51.3 s (normal range: 9.4–12.5 s), or 13% (normal range: 80%–120%), aPTT: 176 s (reference values: 28–40 s), mixing test aPTT: 117 s. FV activity: 0.1%. All other coagulation factors were within normal range. Factor V inhibitor titer was 13.7 Bethesda Units (BU/mL). Acquired factor V deficiency was diagnosed. The routine autoimmune assays performed for systemic autoimmune diseases (including antinuclear antibody (ANA), anti-PCNA antibody, anti-dsDNA antibody, ENA (Extractable Nuclear Antigen) panel, anti-mitochondrial antibody (AMA); anti-smooth muscle antibody, Anti-cytoplasmic antibody (ANCA) panel, anti-MPO and anti-PR3 antibodies, LKM (Liver-Kidney Microsomal) antibody, anti-centromere antibody) yielded negative results, and there was no evidence of lupus anticoagulant or anti-cardiolipin antibodies. During the course of the disease, there were no clinical signs indicative of systemic autoimmune disease. C-Reactive Protein (CRP) was 135 mg/L (normal range: below 10 mg/L) at admission. Subsequent treatments, FV activity and inhibitor titers are presented in Figure 1. The time course of changes in PT, aPTT, FV inhibitor level (Bethesda Inhibitor Assay) and FV activity during the whole course of the disease is shown in Figures 2A–D, respectively.

Bypassing agents successfully controlled bleeding. The patient was first treated with recombinant activated FVII (rFVIIa; 90 μg/kg). After stopping the bleeding, we switched the hemostatic therapy to activated prothrombin complex concentrate (aPCC), also known as Factor eight inhibitor bypassing activity (FEIBA; 100U/kg). Immunosuppressive therapy was initiated with dexamethasone (40 mg/week, for 4 weeks) and rituximab (100 mg/week, for 4 weeks).

On 4th March 2014 the patient developed abdominal pain and anuria associated with a large pelvic hematoma. An emergency operation was performed to evacuate the hematoma, which compressed the urinary bladder and both ureters. To provide coagulation, rFVIIa was applied effectively.

After the postoperative period immunosuppressive therapy was intensified with the CyDRi protocol (cyclophosphamide 1,000 mg on days 1 and 22, dexamethasone 40 mg on days 1, 8, 15, and 22, and rituximab 100 mg on days 1, 8, 15, and 22) [36]. Due to suboptimal response to treatment, on 20th March 2014 plasmapheresis was performed successfully. On the next 2 days following the plasmapheresis immunoglobulin (60 g/day) was administered. As there was no sign of any improvement in factor V activity and the patient had suffered serious bleeding complication, on 3rd April treatment with cyclosporine was started. On 18th April the patient was discharged from our department without any signs of active bleeding. Immunosuppressive therapy was continued: cyclosporine (2 × 200 mg/day, p.o.), rituximab (100 mg once a week, i.v.), dexamethasone (40 mg once a week; i.v.; from October 2014: 20 mg once a week; i.v.), cyclophosphamide (1,000 mg once a month; i.v.).

The response to therapy was unsatisfactory as on 25th May 2014 the patient had to be emergently admitted to our hematology department due to a hematoma in the region of the left ovary. After rFVIIa, antifibrinolytic therapy (tranexamic acid) and platelet transfusions, the bleeding stopped in 2 days and her condition resolved in 12 days. At this time, sirolimus (1 mg per day, p.o.) was started and rituximab discontinued. She responded to the combined cyclosporin + sirolimus + cyclophosphamide + dexamethasone treatment regimen, with slight and delayed improvement of her coagulation parameters through August 2014 (Figure 1). The measured FV activity constantly remained below 5% until December 2014, when it gradually increased to 8% and then to 10% by February 2015 (Figure 1). Despite the continuation of the immunosuppressive therapy, the FV activity fell again to ∼1% in February 2015, without bleeding symptoms. Based on the previous history of life-threatening hemorrhages, the immunosuppressive therapy was once again modified. In March 2015, cyclosporine treatment was discontinued and the patient was started on bortezomib (2 mg once a week, s.c.; dose was decreased from June 2015 to 1.8 mg once a week, s.c. and from November 2015 1.0 mg once a week s.c.) and the dosage of methylprednisolone was reduced from 100 mg once a week in June 2015 to 40 mg once a week in November 2015. The patient did not respond to the combined bortezomib-sirolimus-methylprednisolone therapy, therefore, all immunosuppression was discontinued by the end of November 2015.

At the end of November 2015 the patient, who had therapeutic amenorrhoea for years (drospirenone and ethinyl estradiol as contraception), presented with persistent breakthrough gynecological bleeding. Therefore, the immunosuppressive regimen was once again started with mycophenolate mofetil (500 mg/day p.o.) and dexamethasone (20 mg once a week; i.v) with maintenance sirolimus. Within 2 weeks, the patient showed a marked clinical improvement, and the bleeding stopped. From December 2015, the patient was transitioned to methylprednisolone (40 mg once a week, p.o. alternating with 80 mg once a week, p.o.) while treatment with the sirolimus-mycophenolate mofetil was continued. Due to an unsatisfactory response to therapy (Figure 1), steroid treatment was discontinued in February 2016. The sirolimus-mycophenolate mofetil therapy was discontinued in June 2016. The patient was closely monitored for bleeding events and coagulation status. Her FV activity remained in the range of 1%–2%.

The possibility of allogeneic hematopoietic stem cell transplantation was considered. However, the only sibling of the patient was not human lymphocyte antigen (HLA)-identical. A matched unrelated donor (MUD) transplant was considered, but due to its morbidity and the symptom-free status of the patient at that time, MUD transplantations was not pursued further. The patient continued to be monitored closely for bleeding events and coagulation status. In November 2017, the patient exhibited partial spontaneous remission (Figure 2). By January 2018 her FV activity reached 23% without any treatment. From then on, although the FV activity showed periodical changes, it always remained higher than 8%.

In October 2019, excision of a histologically benign naevus (3 mm in diameter) from the umbilical region was performed. The FV activity of the patient was 8%. This minor surgical procedure was uncomplicated and was performed without the use of a bypassing agent. In the subsequent years the coagulation status of the patient continuously showed gradual spontaneous improvement. Her FV activity was 20% in January 2020 and reached 33% in February 2021 (Figure 2).

The patient became pregnant in March 2021. Her coagulation status was closely monitored. During pregnancy her coagulation parameters normalized (Figure 2). Thus, prophylaxis with a bypassing agent was not indicated to for childbirth. In December 2021, at 39 weeks of gestation the patient gave birth to a healthy child by Caesarean section. The procedure was uncomplicated and there were no bleeding complications during the *postpartum* period. Of note, the patient even received a prophylactic dose of low molecular weight heparin (LMWH; enoxaparine; 4000 IU daily s.c.) for several days in the obstetrics ward according to the obstetric protocol. At present, the coagulation status of the patient is monitored continuously. She is currently asymptomatic and her coagulation parameters are within normal range.

## Discussion

The cause triggering the development of factor V inhibitors in AFVI patients remains largely unknown. Several mechanisms have been proposed, including spontaneous development of autoantibodies, production of antibodies against the bovine factor V that cross-react with human factor V, and alloantibodies [16, 37–40]. In our patient development of AFVI was spontaneous, manifested at the time of surgical intervention for an ovarian cyst that might already have become symptomatic from a hemorrhage.

Many cases of AFVI are iatrogenic, associated with surgical procedures involving the use of topical bovine thrombin glue [4, 16]. Due to the manufacturing procedure, some batches of topical bovine thrombin glue can become contaminated by factor V [41], which can potentially trigger the production of antibodies cross-reacting with human factor V. In our case, the exposure to bovine thrombin during the procedure could not be verified. Iatrogenic AFVIs usually manifest 7–10 days postoperatively and then often disappear within 8–10 weeks. In contrast, non-iatrogenic AFVIs usually persist for significantly longer periods, as it did in our patient. Because bovine thrombin-containing products have been largely replaced since the approval of the less immunogenic recombinant human thrombin by the U.S. Food and Drug Administration in 2007, the incidence of even previously rare iatrogenic AFVI cases has further declined.

Drug-induced development of AFVI has been well documented in the scientific literature [9, 26, 29, 34]. Many of these cases of AFVI are caused by antibiotic (e.g., aminoglycosides [especially streptomycin], β-lactam antibiotics, tetracyclines, and fluoroquinolones [especially ciprofloxacin]) therapy [9, 29, 34]. In our patient a link between medication and development of AFVI could not be established.

The pathogenic role of infections in the development of AFVIs has also been proposed in the literature [1, 6, 7]. Our patient had no symptoms of an infection.

It should be noted that the surgical procedure that triggered the development of AFVI was laparoscopic ovarian cystectomy. Ovarian cysts are known to contain a wide array of antigenic proteins that, theoretically, could also contribute, if released during surgery, to the induction of an autoimmune process. In support of this concept, a causal link between ovarian cysts and autoimmune hemolytic anemia has been documented in the literature [42–45]. The available evidence suggests that cross-reactivity between antigens present in the cyst and red blood cell antigens and consequential production of IgG autoantibodies underlie the pathogenesis of autoimmune hemolytic anemia in patients with ovarian cysts [44]. We cannot rule out a similar mechanism contributing to the pathogenesis of AFVI in our patient.

Systemic autoimmune disorders and malignant diseases underlie the development of AFVIs in approximately 10–20 percent of the cases, respectively. Yet, in our patient neither systemic autoimmune disease nor malignancies were diagnosed during over 7 years of follow-up.

In addition to antibodies neutralizing factor V, clearance-facilitating autoantibodies directed against factor V can also be present in patients with acquired factor V deficiency [28, 46]. Theoretically, it is possible that neutralizing antibodies and clearance-facilitating antibodies are simultaneously present in a patient. Thus, it is possible that test results for AFVIs (Bethesda titer) may become negative if a patient only has clearance-facilitating antibodies. This may contribute to an apparent dissociation of the time course of changes in FV inhibitor level, FV activity and INR/APTT if the cellular sources of these antibodies are differentially affected during the course of treatment.

### Control of symptomatic bleeding

Hemorrhages occur in over two-thirds of patients with AFVI [33]. According to the FV literature, prolongation of PT-INR and aPTT are better predictors of bleedings than the FV inhibitor titer [33]. Our patient also tended to develop major hemorrhages when her FV activity was the lowest and prolongation of PT-INR and aPTT was the most pronounced. Bleeding from mucous membranes are the most common, whereas intracranial hemorrhages [47] and retroperitoneal bleeds associate with the highest mortality. Our patient developed multiple hemorrhagic events, which included gastrointestinal and gynecological bleedings and the formation of a pelvic hematoma—in addition to the peri-operative bleeding events. In patients with AFVI the mortality rate from bleeding was reported to be 12%–15% [33]. Thus, effective control of symptomatic bleedings is the critical first step in the management of AFVI.

Before the diagnosis of AFVI, our patient received FFP and red blood cell transfusion, which turned out to be ineffective. FFP is thought to be ineffective in treatment of AFVI [48] as it has low levels of FV. After diagnosis, hemostasis was obtained by rFVIIa administration for each bleeding event. Administration of rFVIIa as hemostatic therapy has been generally reported to be successful in AFVI patients with significant hemorrhage [26, 49]. Once bleeding control was achieved by the administration of rFVIIa, the patient was switched to aPCC. In previous cases, aPCC has also been used with success [33]. Additionally, several cases have been reported, in which platelet transfusions were used as first-line treatment to control acute bleeding in patients with AFVI [33, 47, 48]. It has been shown that in humans approximately 20% of FV is derived from platelets, which are relatively unaffected by AFVIs and that in patients with AFVI platelets are the primary source of factor V for hemostasis [50].

### Removal of AFVI and immunosuppressive therapies

The second important step in the treatment of AFVI is to eliminate the inhibitor. A number of treatments to reverse AFVI have been tested and reported in the literature. Corticosteroids are often used with success [31, 33] and in many cases are combined with other immunomodulating medications, including rituximab [51] or cyclophoshamide [19]. Rituximab is used to treat a range of autoimmune diseases, including acquired inhibitors of coagulation (acquired FVIII inhibitor, AFVI [51]). Cyclophosphamide, an alkylating agent remains an important treatment for life-threatening autoimmune diseases. Cyclosporine, a calcineurin inhibitor has been used previously to treat acquired inhibitors of coagulation. In our patient immunosuppressive therapy was started with dexamethasone and rituximab, but the response was insufficient. Thus, the immunosuppressive therapy was also intensified with the addition of cyclophosphamide (CyDRi protocol) and then cyclosporine, but these therapies also failed to achieve reversal of AFVIs.

Previous studies demonstrated that plasmapheresis may be effective in AFVI cases with severe hemorrhage [6, 52]. Therapeutic plasma exchange is known to decrease the circulating levels of each plasma immunoglubulin subclass by ∼70%–80% [53]. Further, previously high-dose intravenous immunoglobulin has also been shown to decrease anti-FV inhibitor titers [54]. The current patient was treated with plasmapheresis followed by high-dose intravenous immunoglobulin, which contributed to a rapid but transient decline in the anti-FV inhibitor titers.

When sirolimus (rapamycin) was added to the immunosuppresive treatment regimen, the clinical condition and coagulation parameters of the patient started to improve. Sirolimus/rapamycin is an inhibitor of mTOR (mechanistic target of rapamycin kinase) and is known to inhibit the activation of both T cells and B cells. Sirolimus/rapamycin has been shown to induce complete reversal in patients with corticosteroid-refractory autoimmune cytopenias (including patients with immune thrombocytopenic purpura), who also failed at least one 2nd-line therapy: cyclosporine or rituximab [55]. When this therapy eventually failed, combination therapy with sirolimus plus corticosteroid plus bortezomib and, subsequently, mycophenolate mofetil was attempted. Bortezomib inhibits the proteasome, promoting apoptosis in plasma cells, and also has T-cell inhibitor effects. Mycophenolate mofetil is a potent immunosuppressant, which acts though blocking inosine monophosphate dehydrogenase, required for guanosine production by lymphocytes. In contrast to encouraging results reported in the literature on successful treatment of AFVI or other acquired coagulopathies [5, 56, 57] our patient did not respond to any of these measures.

An interesting aspect of the current case is the delayed reversal of AFVI after the discontinuation of ineffective immunosuppressive therapies, that took place over several years. There are previous case reports documenting the spontaneous remission of AFVI in patients with different underlying comorbidities [18]. However, there are also reports showing that recurrence of AFVI is possible years after spontaneous remission [58]. AFVI is an autoimmune disease and autoimmune diseases are known to show spontaneous remissions, mediated by largely unknown mechanisms. One important observation is that the current patient experienced a significant, rapid (yet still partial) improvement in FV activity during her pregnancy. During pregnancy, affected women often experience remission of autoimmune diseases [59, 60]. However, after childbirth hormonal and physiological changes are quickly reversed back to normal and immune reconstitution is commonly associated with a flare-up of autoimmune diseases. This did not happen to our patient, but long-term monitoring of the coagulation status and the anti-FV inhibitor remains warranted.

In summary, AFVI is a rare hemostatic disorder caused by antibodies against factor V, resulting in bleeding manifestations of varying severity. Effective control of symptomatic bleeding and elimination of the inhibitor through immunosuppressive therapy are the critical steps in management. While the cause and pathogenesis of AFVI remain largely unknown, several mechanisms have been proposed. While spontaneous remission can occur, recurrence is also possible. The current case highlights the delayed reversal of AFVI after the discontinuation of ineffective immunosuppressive therapies, and the improvement in FV activity during pregnancy. Long-term monitoring of the coagulation status and anti-FV inhibitor is necessary.

## Data Availability

The raw data supporting the conclusion of this article will be made available by the authors, without undue reservation.
